# RSV-Related Thrombocytopenia Associated with Transient Cytogenetic Abnormalities in a Recipient of Umbilical Cord Blood Transplantation

**DOI:** 10.1155/2016/8628507

**Published:** 2016-02-02

**Authors:** Sabita D. Pokhrel, Diane L. Persons, Omar S. Aljitawi

**Affiliations:** ^1^Hematology/Oncology and Blood and Marrow Transplant Program, University of Kansas Medical Center, Kansas City, KS 66160, USA; ^2^Pathology and Laboratory Medicine, University of Kansas Medical Center, Kansas City, KS 66160, USA

## Abstract

Respiratory syncytial virus (RSV) infections are associated with thrombocytopenia. The underlying mechanism of thrombocytopenia in this setting is unknown. Herein, we report a case of RSV-related thrombocytopenia associated with transient cytogenetic abnormalities that occurred following umbilical cord blood transplantation.

## 1. Introduction

Respiratory syncytial virus (RSV) infections are associated with extra pulmonary manifestations including thrombocytopenia [1]. The underlying mechanism of thrombocytopenia in RSV infections is unknown. Herein, we report a case of RSV-related thrombocytopenia associated with transient cytogenetic abnormalities that occurred following umbilical cord blood transplantation.

## 2. Case Description

**Figure 1:**
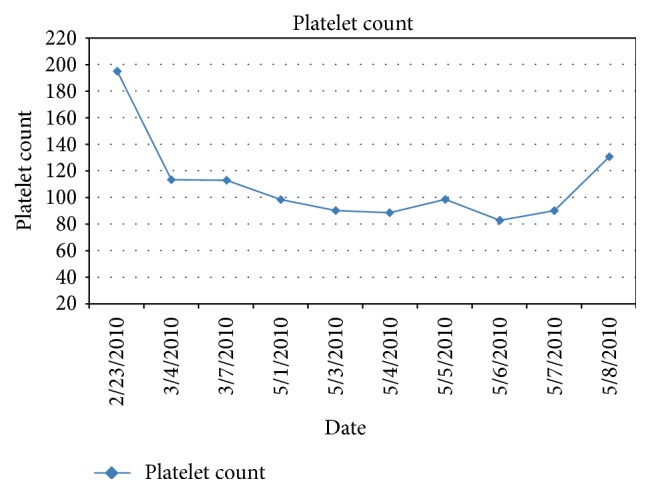
Platelet count values over time.

**Figure 2:**
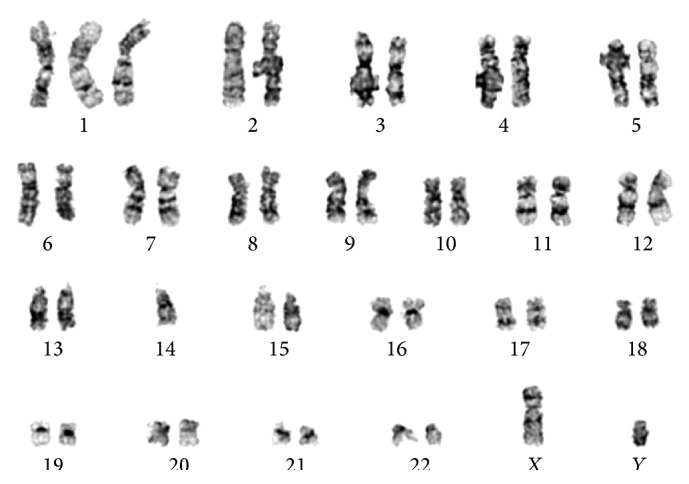
Representative G-banded karyogram showing 46,XY,+1,der(1;14)(q10;q10).

We report here a case of a 28-year-old male patient who underwent a double umbilical cord blood transplant for acute lymphoblastic leukemia (ALL) in second remission in 2008. Original cytogenetic analysis revealed normal karyotype. Nineteen months after transplant, he presented to clinic with upper respiratory infection symptoms. RSV was detected by molecular tests performed on a nasal wash sample and a CT scan of the chest showed right upper lobe cavitary lesion containing a nodular density concerning pneumonia for which he was treated with aerosolized ribavirin. At the same time, the patient was found to be thrombocytopenic (Figure 1). Because of the significant drop in platelet count he underwent an urgent bone marrow evaluation. A new cytogenetic abnormality was detected in two metaphases which showed a derivative chromosome 1 from a translocation between chromosome 1 and chromosome 14 resulting in an extra copy of the long arm of chromosome 1. The final karyotype was 46,XY,+1,der(1;14)(q10;q10)[2]/46,XY[18] (Figure 2). The abnormality was detected in two separate cultures making this finding very suspicious for the presence of abnormal clone. This abnormality was not seen previously and was of concern for a donor driven clonal process as the patient had 100% donor chimerism. The patient was observed with improvement in his platelet count (Figure 2). A follow-up bone marrow was performed 7 months later. Cytogenetics at that time revealed 46,XY[20] with no apparent chromosome abnormalities. Again, he had 100% donor chimerism. The abnormality observed earlier was not detected on follow-up studies and platelets recovered without any intervention (Figure 2). The patient is currently in complete remission with 100% donor chimerism and doing well almost 5 years from the date of detection of the cytogenetic changes.

## 3. Discussion

We report a case of RSV-related thrombocytopenia associated with transient cytogenetic abnormalities that occurred following umbilical cord blood transplantation. Development of thrombocytopenia following transplantation is a sign of possible disease relapse. In our case, the patient had no morphologic changes of leukemia. Donor cells leukemia (DCL), on the other hand, is a rare complication following allogeneic hematopoietic stem cell transplant [2–4]. Wang et al. reported 10 cases of donor cell leukemia in allogeneic hematopoietic stem cell transplant recipients performed between 1991 and 2000 for different hematologic malignancies from 3 different bone marrow transplant centers [3]. Of interest out of 10 cases, 1 case demonstrated a spontaneous remission without pertinent treatment 1.5 years after the diagnosis of donor derived myelodysplastic syndrome. In our case, though the cytogenetic changes were probably of donor origin, there was no evidence of myelodysplastic syndrome or leukemia. On the other hand, Otero et al. [5] reported transient chromosomal rearrangement in three children with ALL after unrelated cord blood transplantation. They were associated with delayed hematological recovery, primary rejection, and autologous reconstitution without disease relapse or secondary malignancy [5]. Our patient did not experience any rejection or autologous constitution.

The most probable explanation for our findings is RSV infection, though no viral etiology was described in the cases reported by Otero et al. The transient cytogenetic changes in our case occurred at the time of thrombocytopenia and accordingly might provide an explanation for RSV-related thrombocytopenia. Recent data suggest that RSV might infect bone marrow stromal cells [6] which might provide the right milieu for such transient cytogenetic changes to occur. Alternatively, thrombocytopenia and cytogenetic abnormalities might have occurred independently. More cases and a longer follow-up are needed to know the real clinical significance of the transient chromosomal rearrangements in patients with RSV following umbilical cord blood transplantation.
